# Biodiversity of Rhizosphere Fungi from *Suaeda glauca* in the Yellow River Delta and Their Agricultural Antifungal and Herbicidal Potentials

**DOI:** 10.3390/md23120460

**Published:** 2025-11-29

**Authors:** Tian-Li Qu, Hong Li, Dong-Fang Cao, Meng-Ya Li, Chen Zhao, Li-Yuan Zhang, Bao-Hua Zhang, Lin Xiao, Feng-Yu Du

**Affiliations:** 1 College of Chemistry and Pharmacy, Qingdao Agricultural University, Qingdao 266109, China; qtli0411@163.com (T.-L.Q.); zhangbaohua76@163.com (B.-H.Z.); 2 College of Plant Health and Medicine, Qingdao Agricultural University, Qingdao 266109, China; li15145880540@163.com (H.L.); cdf0911y@163.com (D.-F.C.); hli569@126.com (M.-Y.L.); zc171703121@163.com (C.Z.); zhangliyuan_2222@163.com (L.-Y.Z.)

**Keywords:** rhizosphere fungi, *Suaeda glauca*, Yellow River Delta, antifungal potentials, herbicidal potentials

## Abstract

*Suaeda glauca* is a typical halophyte distributed in coastal and inland zones of the Yellow River Delta. Its rhizosphere soil is a potential source for exploring various fungi and their metabolites. In this study, the rhizosphere fungal community of *S. glauca* was evaluated with high-throughput sequencing, suggesting that it was tightly associated with seasonal variation and soil physicochemical factors. The fungal diversity at the genus level when sampling in May was better than that in July and October. The physicochemical factors TK and TP exerted relatively positive effects on the fungal diversity, while SOM, pH and TDS exhibited negative ones. Using the dilution plating method, 55 cultivable fungal strains were further isolated from the rhizosphere soil of *S. glauca*, in which *Aspergillus* and *Penicillium* were the dominant ones. A total of 47 and 20 strains showed antifungal and herbicidal activity, respectively. Finally, bioassay-guided isolation from the representative strain *A. tabacinus* GD-25 obtained three polyketides (**1**–**3**) and one diphenyl ether (**4**). **1** (sydonic acid) and **4** (diorcinol) greatly inhibited mycelial vitality of *Bortrytis cinerea*, with IC_50_ values of 75.4 and 67.4 mg/L, respectively. In addition, 50 μg/mL of **4** could almost inhibit seedling growth of *Echinochloa crusgalli*.

## 1. Introduction

The Yellow River Delta wetland is one of the most representative coastal wetlands in China, which belongs to the transitional zone between terrestrial and marine ecosystems [1]. It plays an important role in protecting the biodiversity of the Yellow River and the Bohai Sea, which provides a suitable living environment for various ecological types including animals, plants and microorganisms [2,3]. However, a substantial part of the Yellow River Delta is saline–alkali land with underdeveloped land resources, almost 700,000 hectares [4,5]. Excessive soil salinity has negative effects on microbial communities, soil fertility, crop growth, etc. [6]. Nevertheless, it can provide a good habitat for halophyte and salt-tolerant microorganisms [7].

When halophytes are confronted with saline–alkali stress, their own tolerance mechanisms enable them to adapt to the environment rapidly [8,9]. Rhizosphere microorganisms can promote plant growth, alleviate saline–alkali stress and synergistically regulate soil ecology together with plants [10,11]. *Suaeda glauca* is a representative halophyte widely distributed in the Yellow River Delta. It has the ability to absorb salts and heavy metals in the soil, which is closely related to the soil microorganisms in the rhizosphere of *S. glauca* [12]. Due to its internal and external high-salinity environments, *S. glauca* has been considered a potential source for various bioactive microorganisms, which could produce different interesting secondary metabolites [13,14,15,16,17]. The rational utilization of these microorganisms is extremely important for the development and utilization of saline–alkali land resources [1]. Research on their bioactive potentials has mainly focused on the pharmacological applications, such as anticancer, antibacterial, antioxidant and enzyme-inhibitory effects, but there are few reports on their agricultural bioactive potential [18,19,20,21].

During our ongoing search for biocontrol agents in agriculture [13,14,15,22,23], *S. glauca*-derived fungus has attracted our attention because of its integrated potential against both weed and phytopathogens. In this study, high-throughput ITS sequencing was employed to evaluate the rhizosphere fungal community of *S. glauca* in the Yellow River Delta. The relationship between the fungal community and soil physicochemical factors was also analyzed. Then, cultivable fungi were isolated, identified and evaluated for their agricultural antifungal and herbicidal potentials. Finally, one representative strain was fermented to search for its bioactive metabolites. This study provides a feasible routine for the search of agricultural bioactive fungi from the rhizosphere soil of *S. glauca* and reveals their great potentials for the development of agricultural biocontrol agents.

## 2. Results and Discussion

### 2.1. Biodiversity of Rhizosphere Fungal Community of S. glauca

#### 2.1.1. Rhizosphere Soil Physicochemical Factors

**Figure 1:**
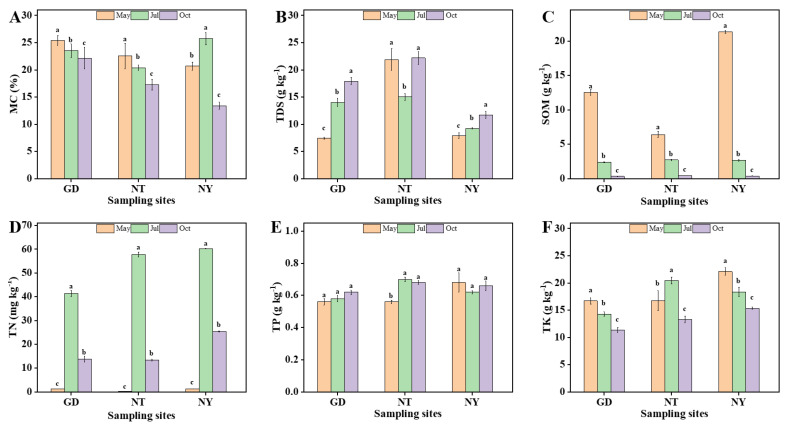
Rhizosphere soil physicochemical factors of *S. glauca* in sampling zones GD, NT and NY of the Yellow River Delta. (**A**) Moisture content (MC), (**B**) total dissolved salt content (TDS), (**C**) soil organic matter (SOM), (**D**) total nitrogen (TN), (**E**) total phosphorus (TP) and (**F**) total potassium (TK). Vertical bars indicate standard errors of the means with three repeated experiments. Columns with different letters indicate significant differences according to Duncan’s multiple range test (*p* < 0.05).

As shown in Figure 1 and Table S1, sampling zone NT showed the highest value of total dissolved salt content (TDS) in three zones. Meanwhile, the content of soil organic matter (SOM) in zone NY in May was significantly higher than those in zones GD and NT (*p* < 0.05). Therefore, *S. glauca* in zone NY could be observed with more dense distribution.

Soil physicochemical factors also exhibited obvious seasonality variations in the three sampling zones GD, NT and NY (Figure 1 and Table S1). The values of electrical conductivity (EC) and TDS in the GD and NY zones markedly enhanced from May to October, while total potassium (TK) and SOM in the GD and NY zones significantly decreased. Moreover, the contents of total nitrogen (TN) in the three zones all reached their peaks in July, while pH values (Figure S1) and moisture content (MC) recorded their minimum values in July and October, respectively.

#### 2.1.2. Rhizosphere Fungal Diversity

**Figure 2:**
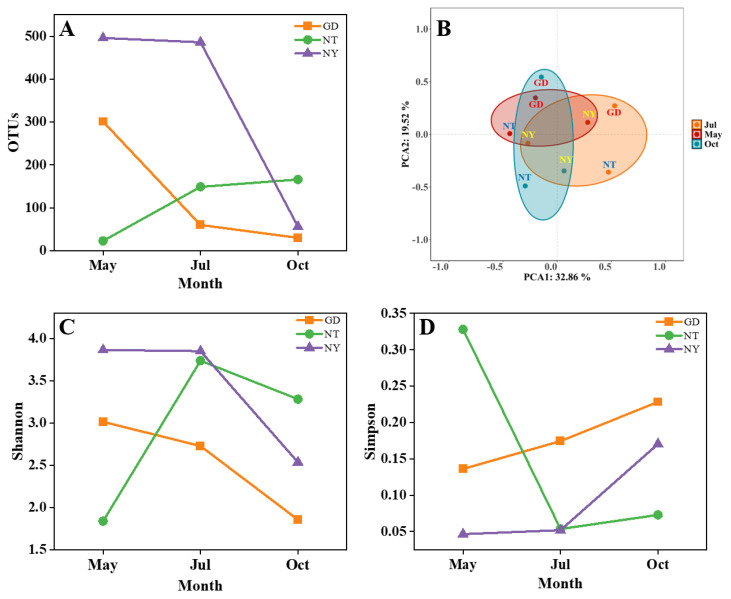
Rhizosphere fungal diversity of *S. glauca* in sampling zones GD, NT and NY of the Yellow River Delta. (**A**) OTUs, (**B**) PCA, (**C**) Shannon diversity index and (**D**) Simpson diversity index.

The rhizosphere fungal diversity of *S. glauca* in sampling zones GD, NT and NY has been evaluated using the methods of principal component analysis (PCA) and alpha-diversity analysis. As suggested in Figure 2A, zone NY showed significantly more operational taxonomic units (OTUs) than zones GD and NT in May and July. PCA results (Figure 2B) suggested that the rhizosphere fungal communities in the three sampling zones were obviously different. In addition, the seasonal transition from May (spring) to July (summer) and October (autumn) could also greatly affect rhizosphere fungal diversity.

Simpson, Shannon, Chao1 and Ace indices were further calculated to evaluate alpha-diversity of rhizosphere fungi in the three sampling zones. Analyses of Shannon (Figure 2C) and Simpson parameters (Figure 2D) indicated that the fungal diversity of zone NY was significantly higher than that of zones GD and NT in May, then very close to NT in July, and finally lower than NT in October. Moreover, zone GD showed lower fungal diversity than zones NY and NT in July and October. Chao1 and Ace indices also suggested similar results to those of the Simpson and Shannon ones (Table S2).

#### 2.1.3. Rhizosphere Fungal Community Structure

**Figure 3:**
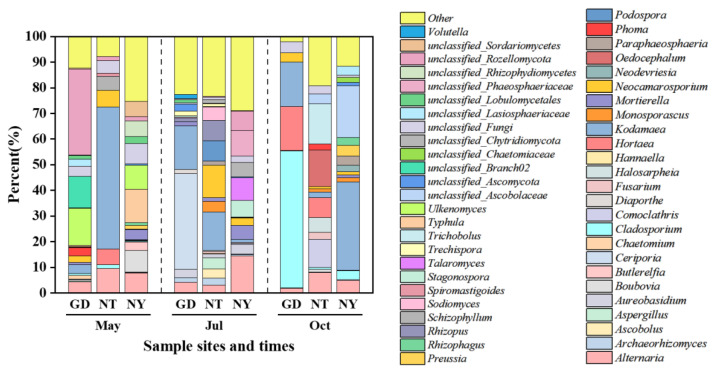
Rhizosphere fungal community structure (genera with relative abundance ranking in top 30) of *S. glauca* in sampling zones GD, NT and NY of the Yellow River Delta.

The rhizosphere fungal community structure of *S. glauca* in sampling zones GD, NT and NY was further analyzed at the genus level. As demonstrated in Figure 3, the fungal community structure exhibited obvious seasonal and locational variability. From the perspective of seasonal difference, the genus diversity when sampling in May (spring) was slightly higher than that in July (summer), and significantly better than that in October (Autumn). *Ceriporia* (12.44%), *Kodamaea* (11.06%) and *Alternaria* (7.20%) were the dominant genera in July, while *Kodamaea* was also the superior genus in May and October, with a relative abundance of 19.64% and 18.01%, respectively. *Cladosporium* was 19.16% in October, which was slightly higher than that of *Kodamaea*. Moreover, *Alternaria* was also the main genus in May and October, with the relative abundances both above 5%.

In terms of different sampling zones, the genus diversity of zone NY was higher than that of zones GD and NT. Take July as an example; except for unclassified species, genera in zone NY with a relative abundance above 5% were *Alternaria* (14.41%), *Talaromyces* (8.85%), *Stagonospora* (6.51%) and *Mortierella* (5.26%), while in zone NT, they were *Kodamaea* (15.02%), *Neocamarosporium* (12.75%), *Rhizopus* (7.96%) and *Podospora* (7.88%). For zone GD, *Ceriporia* and *Kodamaea* were two dominant genera with a total abundance above 50%.

#### 2.1.4. Relationship Between Fungal Diversity and Soil Physicochemical Factors

**Figure 4:**
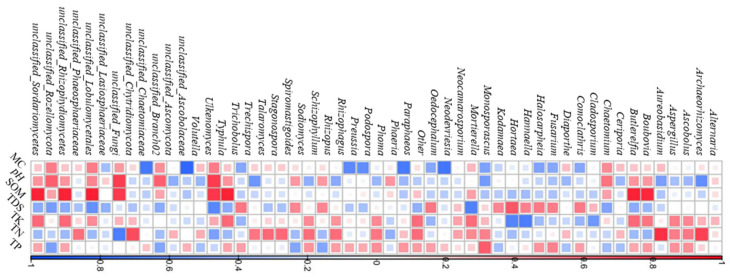
Relationship between dominant genera of rhizosphere fungi and soil physicochemical factors (red suggests a positive correlation, while blue indicates a negative one).

As shown in Figure 4, the impact of different soil physicochemical factors on the fungal relative abundances varied significantly. For example, the genera *Archaeorhizomyces* and *Aureobasidium* showed a greatly positive correlation with soil TN, but a significantly negative one with pH. Meanwhile, the genera *Kodamaea* and *Alternaria* were not obviously affected by soil physicochemical factors, and they therefore became the dominant ones in the three sampling zones (Figure 3).

TK and TP were representative factors which showed more positive correlations with dominant fungi at proportions of 64% and 56%, respectively, while SOM, pH and TDS exhibited more negative ones at proportions of 62%, 62% and 56%, respectively (Figure 4). This result suggested that TK and TP exerted relatively positive effects on fungal diversity in the three sampling zones, while SOM, pH and TDS exhibited negative ones. It was also found that TP was an important factor which could positively affect fungal number and biomass in the macrophyte rhizosphere [24]. Meanwhile, Refs. [25,26] reported that the content of SOM generally showed a negative correlation with fungal community structures. One possible interpretation for the negative relationship of SOM was that the interspecific competition for SOM would result in the survival of only fewer dominant highly competitive fungi, and therefore a decrease in fungal diversity [27]. On the other hand, the fungal decomposition ability of SOM increased with its community diversity, consequently resulting in SOM depletion [28].

### 2.2. Biodiversity of Cultivable Rhizosphere Fungi of S. glauca

#### 2.2.1. Isolation and Identification of Cultivable Rhizosphere Fungi

A total of 55 de-duplicated fungal strains were isolated and identified from the rhizosphere soil of *S. glauca* in the three sampling zones (Tables S3 and S4). The fungal number and diversity when sampling in May were 31 strains distributed in 13 genera, which were higher than those in July and October (Table S3). This result is consistent with the analysis of fungal community structure in Section 2.1.3. The isolated fungal strains belonged to 20 genera, of which *Aspergillus* and *Penicillium* were dominant genera, accounting for 30.9% and 16.3%, respectively (Table S3). These results were not entirely in agreement with the fungal diversity on the basis of the high-throughput ITS sequencing, which might be related to non-cultivability or slow growth of a considerable number of fungi in the PDA medium.

#### 2.2.2. Agricultural Antifungal and Herbicidal Potentials of Isolated Fungi

**Figure 5:**
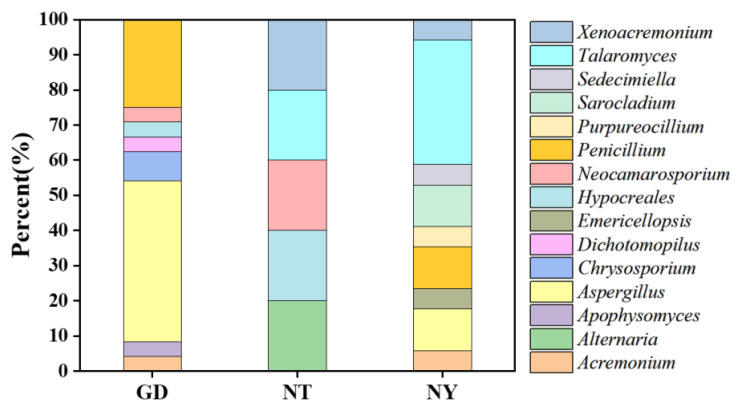
Classifications at the genus level of antifungal rhizosphere fungi from sampling zones GD, NT and NY.

**Figure 6:**
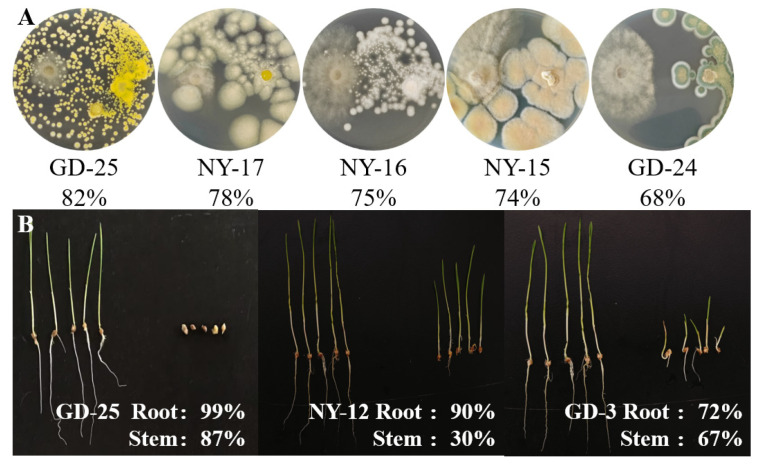
Antifungal and herbicidal potentials of representative fungal strains. (**A**) Antifungal activities against *B. cinerea* using the dual-culture method. (**B**) Herbicidal effects against *E. crusgalli* using the small cup method.

As shown in Table S4, 47 fungal strains (85.5%) showed antifungal potentials against *Botrytis cinerea* CJ-8 using the dual-culture method. The fungal diversity in zone GD was 25 strains distributed in nine genera, which was higher than that in NT (6 strains in five genera) and NY (17 strains in nine genera) (Table S4 and Figure 5). Strains *Aspergillus* and *Penicillium* were representative ones in zone GD, accounting for 48% and 24%, respectively. Meanwhile, in the NY region, *Talaromyces* was the dominant genus, accounting for 35.3%. As suggested in Table S4 and Figure 6, there were five representative antifungal strains with inhibition rates above 65%, including *A. tabacinus* GD-25, *P. brasilianum* NY-17 and NY-15, *Sarocladium terricola* NY-16 and *P. oxalicum* GD-24.

Compared to antifungal strains, only 20 fungal ones (36.4%) exhibited herbicidal potentials against seedling growth of *Echinochloa crusgalli* (Table S4). These strains belong to 10 genera, of which *Aspergillus* was the dominant one with a proportion of 35%. *A. tabacinus* GD-25 and *A. iizukae* NY-12 showed significant herbicidal effects against the root growth of *E. crusgalli* seedlings, with inhibition rates of 99.3% and 90.0%, respectively (Figure 6). However, other fungal strains only exhibited medium or weak herbicidal potentials against *E. crusgalli* (Table S4).

### 2.3. Bioassay-Guided Isolation of A. tabacinus GD-25

**Figure 7:**
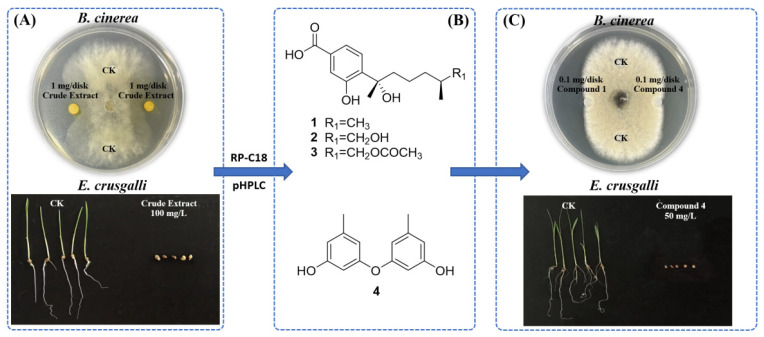
Bioassay-guided isolation of *A. tabacinus* GD-25. (**A**) Antifungal and herbicidal activities of the crude extract of *A. tabacinus* GD-25 against *B. cinerea* CJ-8 and *E. crusgalli*, respectively. (**B**) Structures of bioactive compounds **1**–**4**. (**C**) Antifungal potentials of compounds **1** and **4** against *B. cinerea* CJ-8 and herbicidal activity of **4** against *E. crusgalli*.

The crude extract of *A. tabacinus* GD-25 showed significant antifungal and herbicidal potentials against *B. cinerea* CJ-8 and *E. crusgalli*, respectively (Figure 7A). Its further bioassay-guided isolation yielded four bioactive metabolites, including three polyketides (**1**–**3**) and one diphenyl ether (**4**) (Figure 7B). Their structures were determined by detailed analyses of their ^1^H and ^13^C nuclear magnetic resonance (NMR) spectra (Figures S2–S9), as well as comparisons with previously published reports as follows: sydonic acid (**1**) [29], 12-hydroxy sydonic acid (**2**) [29], 12-acetoxysydonic acid (**3**) [30] and diorcinol (**4**) [31]. Compounds **1**–**3** were first isolated from the species of *A. tabacinus*.

Compounds **1** and **4** exhibited great antifungal potentials against the mycelial growth of *B. cinerea* CJ-8 (Figure 7C), while **2** and **3** only showed weak ones. Further antifungal quantitative evaluation using the resazurin-coloration method suggested that **1** and **4** greatly inhibited mycelial vitality of *B. cinerea*, with IC_50_ values of 75.4 and 67.4 mg/L, respectively, better than 234.3 mg/L for the commonly used fungicide boscalid. Preliminary structure–activity analysis of polyketides **1**–**3** indicated that the methyl group of R_1_ should be related to the antifungal potential of **1**. Very recently, it had also been reported that diorcinol (**4**) could effectively reduce the development of rice blast, tomato late blight and pepper anthracnose [32]. The herbicidal component of *A. tabacinus* GD-25 was only compound **4**, which could almost inhibit seedling growth of *E. crusgalli* at a concentration of 50 μg/mL (Figure 7C). Its EC_50_ value against the radicle growth of *E. crusgalli* seedlings was only 7.9 μg/mL. To our best knowledge, this is the first report of antifungal potential of **1** and herbicidal activity of **4**.

## 3. Materials and Methods

### 3.1. Description of Sampling Sites

**Figure 8:**
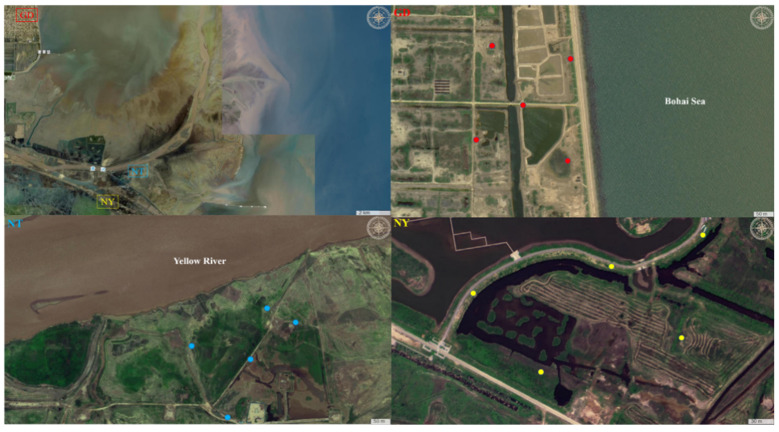
Aerial views of the three sampling zones in the Yellow River Delta wetland (GD, red; NT, blue; NY, yellow). (GD) The intertidal zone near the Bohai Sea, (NT) the transition zone near the Yellow River and (NY) the inland zone. Maps source: National Geographic Information Public Service Platform (https://www.tianditu.gov.cn/, China), Access date: February 25, 2025.

As shown in Figure 8, three representative sampling zones GD, NT and NY were chosen in the Yellow River Delta wetland. Their location information is as follows: GD zone at N37°86′ and E119°10′ near the Bohai Sea, NT at N37°76′ and E119°19′ adjacent to the Yellow River, and NY at N37°74′ and E119°16′. To avoid local deviations affecting the results, based on the principles of random sampling and uniform distribution, the five-point sampling method was used to collect rhizosphere soil of *S. glauca* in the three sampling zones in May, July and October 2023, respectively. The three selected months can accurately capture the responses of community diversity at the three locations to seasonal changes. The brief procedure is as follows: Five sampling points (1 m × 1 m) were set up approximately 300 m apart from each other to ensure ecological segregation. The whole plant of *S. glauca* was excavated with rhizosphere soil, which was then collected into a sterile sampling bag. Soils of the same sampling zones were thoroughly mixed for further analyses of physicochemical factors and the fungal community.

### 3.2. High-Throughput Sequencing and Analysis of Fungal Community Diversity

Soil samples were pretreated with liquid nitrogen and then sent to Sangon Biotech (Shanghai) Co., Ltd. (Shanghai, China) for high-throughput sequencing. The sequencing platform adopts Illumina NovaSeq 6000 (Beijing Biomarker Technology Co., Ltd., Beijing, China) [33]. After the data were returned, PCA, alpha-diversity (OTUs, ACE, Chao1, Simpson and Shannon) and fungal community structure analyses were conducted as previously described [34,35].

### 3.3. Soil Physiochemical Factors

Soil physicochemical factors were analyzed based on standard methods. Rhizosphere soil MC [36] was determined on the basis of the water loss between oven-dried soil (105 °C for 48 h) and wet soil. The pH [37] and electrical conductivity (EC) values were measured using a pH meter and an electrical conductivity meter, respectively. After determining the EC value, the total dissolved salt content (TDS) was calculated by converting the standard curve. SOM was quantified using the potassium dichromate oxidation method with external heating. TN was examined by a Kjeldahl apparatus (Kjeltec™8000, FOSS Group Co., Hillerød, Denmark). For soil TP and TK, another 0.25 g soil sample was extracted and acidified in turn using HCl, HNO_3_ and HF, and then analyzed by ICP (OPTMA8000DV, Inductively Coupled Plasma–optical emission spectroscopy, PerkinElmer Enterprise Management Co., Ltd., Waltham, MA, USA).

### 3.4. Isolation of Fungal Strains from S. glauca Rhizosphere Soil

Fungal strains from *S. glauca* rhizosphere soil were separated using the dilution plating technique [38]. First, 10 g of soil sample and 90 mL of sterile water were added to a 250 mL shaker flask with glass beads and fully suspended at 160 rpm for 1 h. Subsequently, serial 10-fold dilutions were performed, and 100 µL aliquots of 10^−1^ and 10^−2^ dilutions were uniformly coated on the surface of a PDA plate (containing 3% sea salt). All plates were incubated at 28 °C for 3–4 days to observe the appearance of fungal colonies, which were further purified by transferring a small amount of mycelia from the colony edge to a new PDA plate [39].

### 3.5. Identification of Fungal Strain

The total genomic DNA of the strain was extracted using the B518229 fungal genomic DNA rapid extraction kit. The ITS region was amplified using ITS1/ITS4 primers, and the target gene sequence was obtained by Sanger sequencing (sequencing was completed by Sangon Biotech (Shanghai) Co., Ltd.). Based on the determined ITS sequence, blast comparative analysis was conducted in NCBI to establish a phylogenetic tree for the preliminary identification of strains.

### 3.6. Antifungal and Herbicidal Evaluations

The antifungal activity was determined by the dual-culture method. The isolated fungi and pathogen *B. cinerea* (CJ-8) were symmetrically placed on both sides of the PDA plate and then cultured at 25 °C for 3–5 days. The inhibition rates were calculated as described previously [13,40].

The isolated fungi were fermented on solid rice media at 28 °C for 30 days, which were further extracted with ethyl acetate to obtain crude extracts. Their herbicidal potentials were determined using the small cup method [41]. The germinated *E. crusgalli* seeds were planted in agar beakers with 100 mg/L crude extracts and then cultured at 28 °C for 3–5 days. The inhibition rates of roots and stems were calculated, and the experiment was conducted in three parallel repeats.

### 3.7. Fermentation, Extraction and Bioassay-Guided Isolation of A. tabacinus GD-25

The fungus *A. tabacinus* GD-25 was statically fermented at 28 °C for 30 days on the solid rice medium, which was conducted in 10 × 1 L conical flasks containing rice (100 g/flask), peptone (0.6 g/flask) and natural seawater (100 mL/flask). The fungal culture was exhaustively extracted using ethyl acetate (EtOAc) to obtain a crude extract (12.2 g), which was fractionated via column chromatography over RP-C_18_ eluting with a MeOH–H_2_O gradient (from 1:9 to 1:0) to yield six subfractions (Fr. 5-1 to 5-6). Bioactive Fr. 5-3 was purified using semi-pHPLC (30% MeCN–H_2_O, 3 mL/min) to obtain compounds **1** (35.9 mg, t*_R_* = 19.3 min), **2** (6.8 mg, t*_R_* = 15.9 min), **3** (5.6 mg, t*_R_* = 17.4 min) and **4** (23.7 mg, t*_R_* = 22.8 min). The methods of disk diffusion [25,26] and resazurin coloration [40,42] were used to qualitatively and quantitatively evaluate antifungal potentials of the crude extract and its isolated fractions and compounds, respectively, while their herbicidal activities against *E. crusgalli* were determined using the same method as described in 3.6.

## 4. Conclusions

The present study analyzed the community structure of rhizosphere fungi from *S. glauca* in the Yellow River Delta and further evaluated their agricultural antifungal and herbicidal potentials. The main findings included the following: (1) The community structure of rhizosphere fungi significantly varied with seasonal turnover and soil physicochemical factors. The fungal diversity at the genus level when sampling in May was better than that in July and October. Some factors, including TK, TP, SOM, pH and TDS, were important for the mediation of fungal community composition. (2) The cultivable rhizosphere fungi from *S. glauca* belonged to 20 genera, of which *Aspergillus* and *Penicillium* were dominant genera, accounting for 30.9% and 16.3%, respectively. (3) Of a total of 55 cultivable fungal strains, 47 strains showed antifungal potentials and 20 strains showed herbicidal ones. (4) The metabolites sydonic acid (**1**) and diorcinol (**4**) from *A. tabacinus* GD-25 showed significant antifungal and herbicidal potentials. These findings undoubtedly provide the insight that rhizosphere fungi of *S. glauca* possess distinct microbial diversity and communities, which are a rich reservoir of microorganisms with potential agricultural antifungal and herbicidal properties.

## Data Availability

The research data are available in the supporting information.

## Supplementary Materials

The following supporting information can be downloaded at https://www.mdpi.com/article/10.3390/md23120460/s1. Figure S1: Rhizosphere soil pH values of *S. glauca* in sampling zones GD, NT and NY of the Yellow River Delta; Figure S2: ^1^H NMR (500 MHz, CD_3_OD) spectrum of compound **1**; Figure S3: ^13^C NMR (125 MHz, CD_3_OD) spectrum of compound **1**; Figure S4: ^1^H NMR (500 MHz, CD_3_OD) spectrum of compound **2**; Figure S5: ^13^C NMR (125 MHz, CD_3_OD) spectrum of compound **2**; Figure S6: ^1^H NMR (500 MHz, CD_3_OD) spectrum of compound **3**; Figure S7: ^13^C NMR (125 MHz, CD_3_OD) spectrum of compound **3**; Figure S8: ^1^H NMR (500 MHz, CD_3_OD) spectrum of compound **4**; Figure S9: ^13^C NMR (125 MHz, CD_3_OD) spectrum of compound **4**; Table S1: Soil physicochemical factors of three sampling zones; Table S2: Alpha-diversity indices of three sampling zones; Table S3: Isolation of cultivable rhizosphere fungi from the three sampling zones and their classifications at the genus level; Table S4: Identification of cultivable rhizosphere fungi from three sampling zones and their antifungal and herbicidal potentials.
