# Supplementary material for: Biodiversity of Rhizosphere Fungi from Suaeda glauca in the Yellow River Delta and Their Agricultural Antifungal and Herbicidal Potentials
Source: Mar Drugs. 2025 Nov 29;23(12):460. doi: 10.3390/md23120460 (PMC12735306; doi:10.3390/md23120460)
Supplement: Supplementary file 1 [file marinedrugs-23-00460-s001.zip › marinedrugs-4003841-supplementary.pdf]

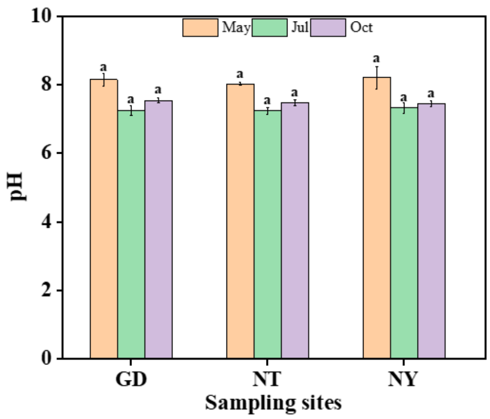

Figure S1. Rhizosphere soil pH values of *S. glauca* in sampling zones GD, NT and NY of the Yellow River Delta.

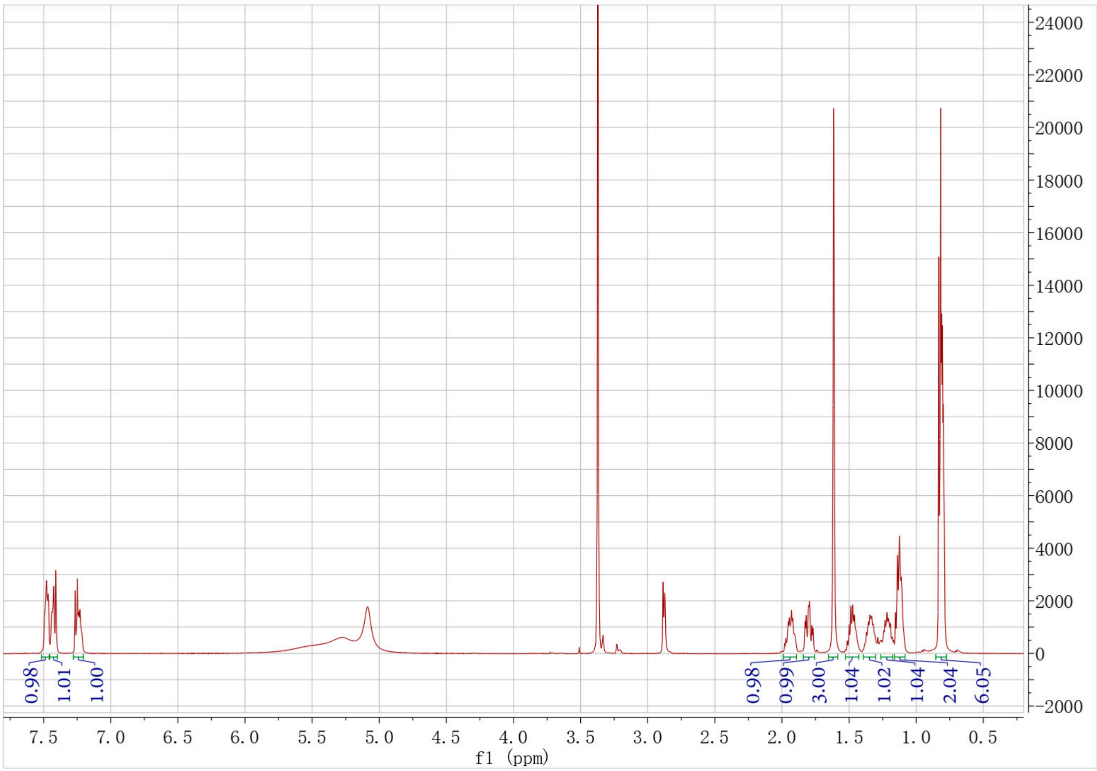

Figure S2. ^{1}H NMR (500 MHz, CD_3OD) spectrum of compound **1**.

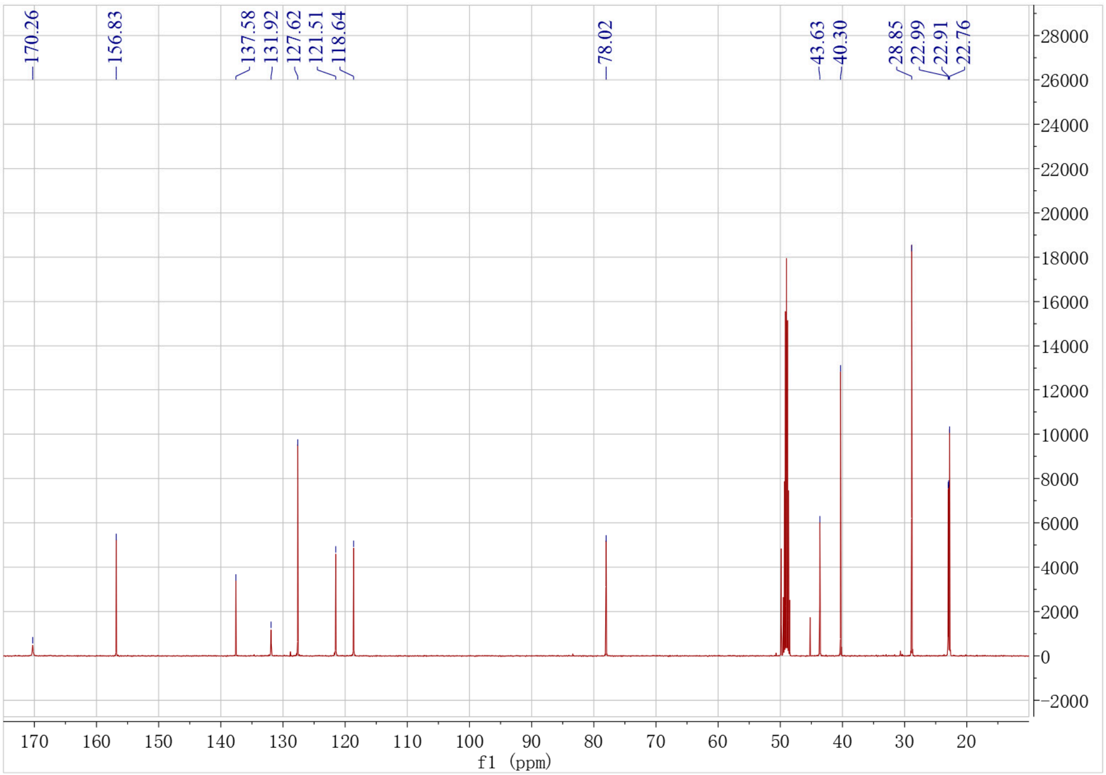

Figure S3. ^{13}C NMR (125 MHz, CD_3OD) spectrum of compound **1**.

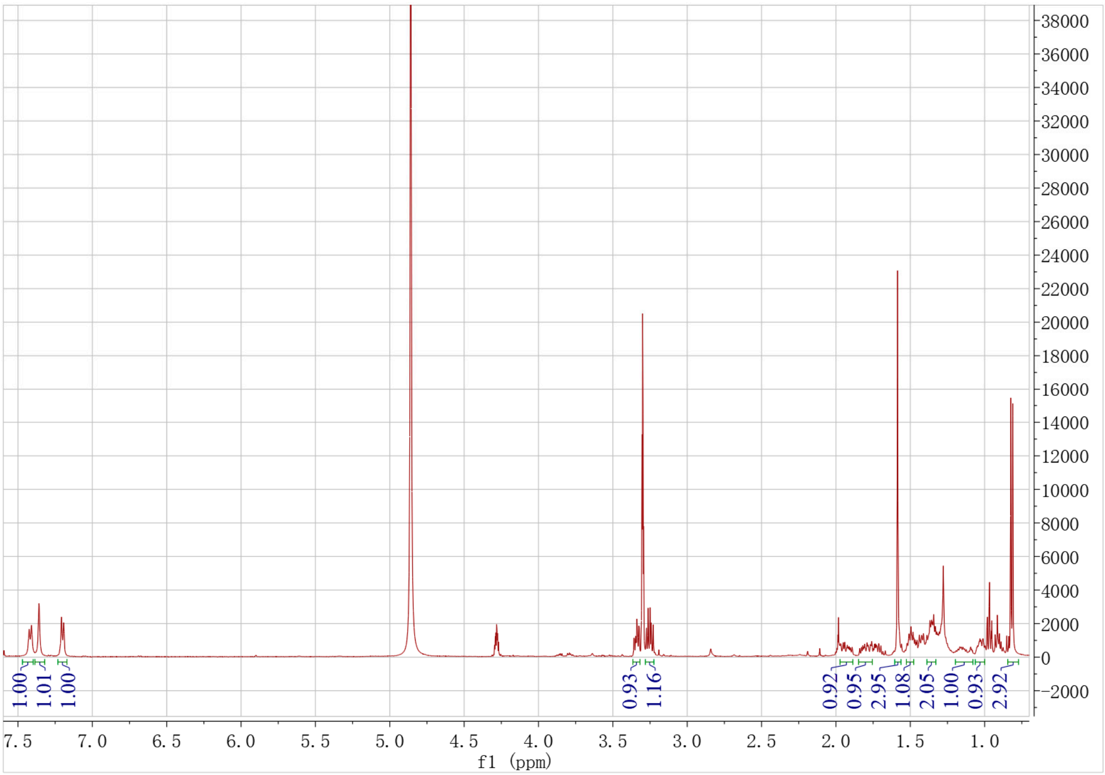

Figure S4. ^{1}H NMR (500 MHz, CD_3OD) spectrum of compound **2**.

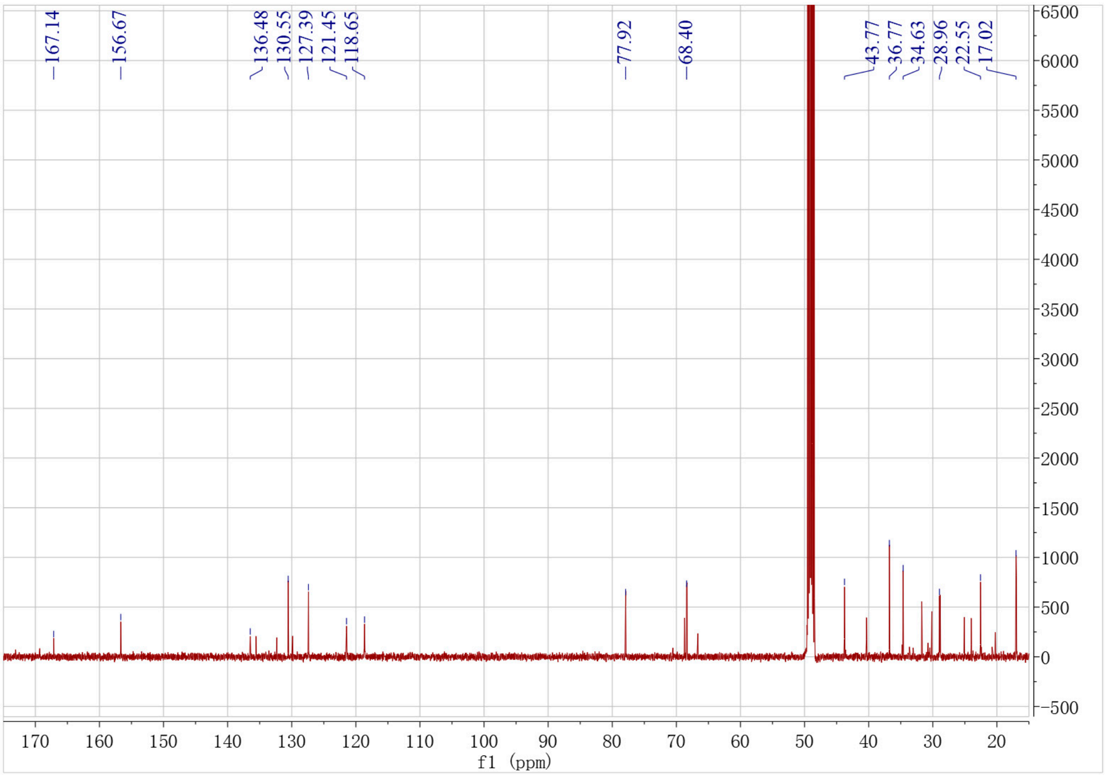

Figure S5. ^{13}C NMR (125 MHz, CD_3OD) spectrum of compound **2**.

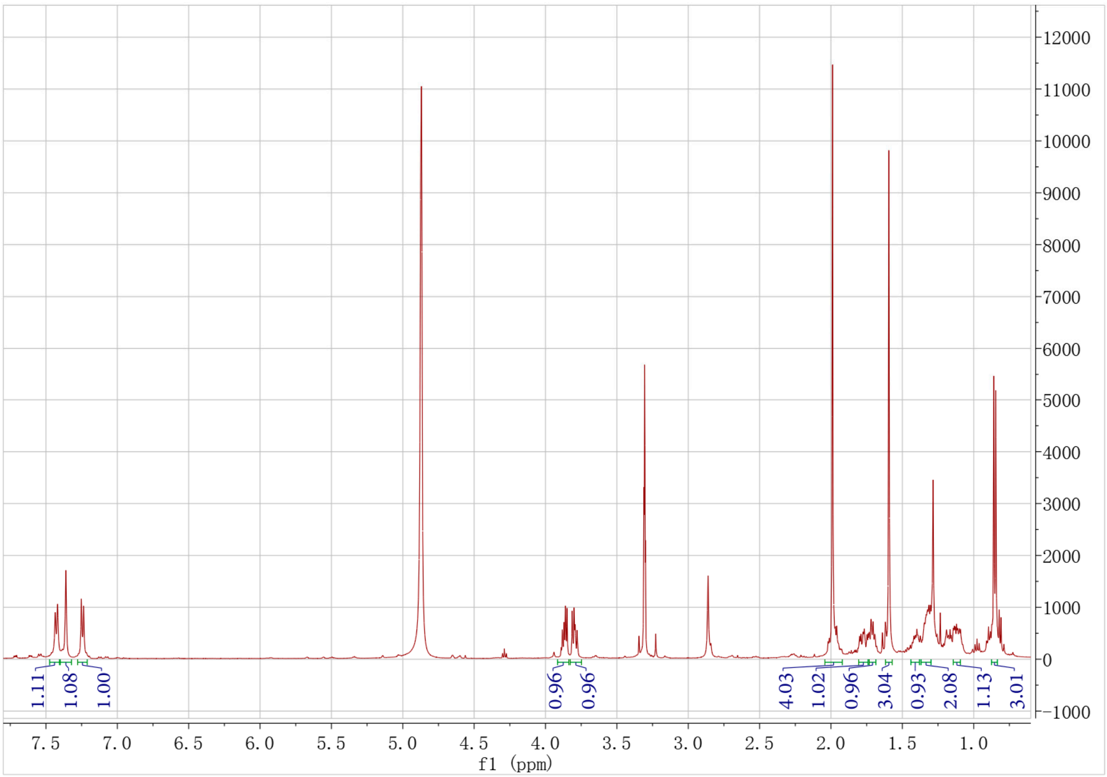

Figure S6. ^{1}H NMR (500 MHz, CD_3OD) spectrum of compound **3**.

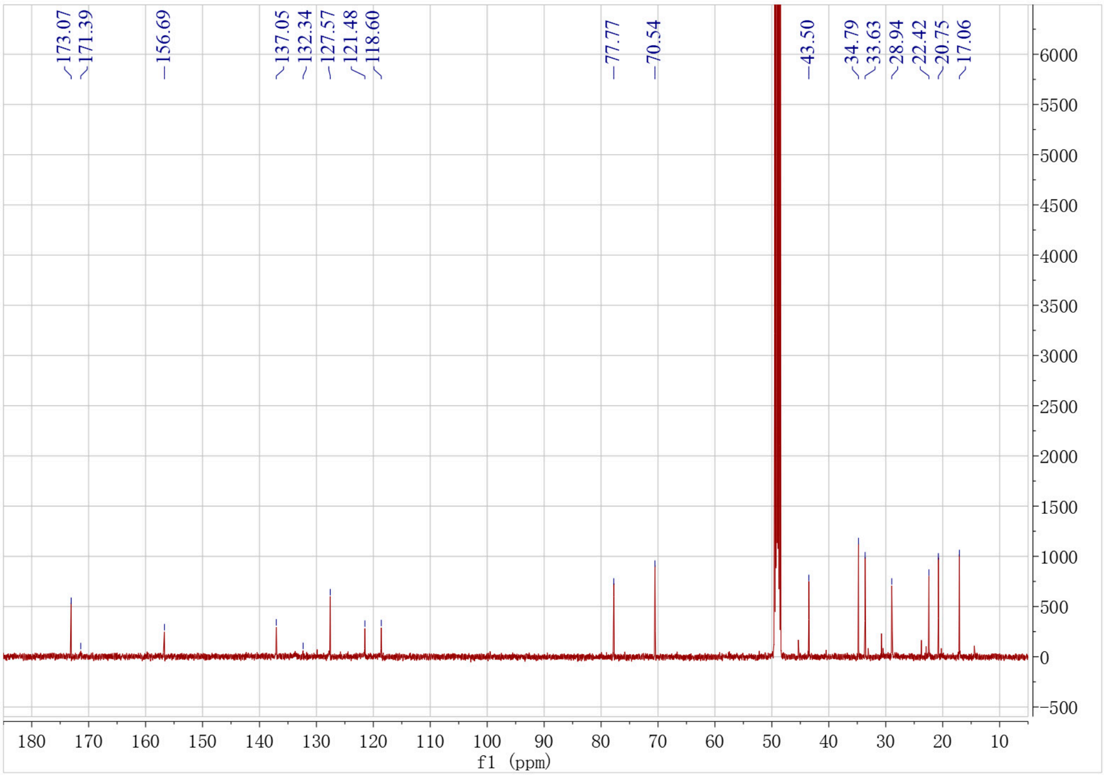

Figure S7. ^{13}C NMR (125 MHz, CD_3OD) spectrum of compound **3**.

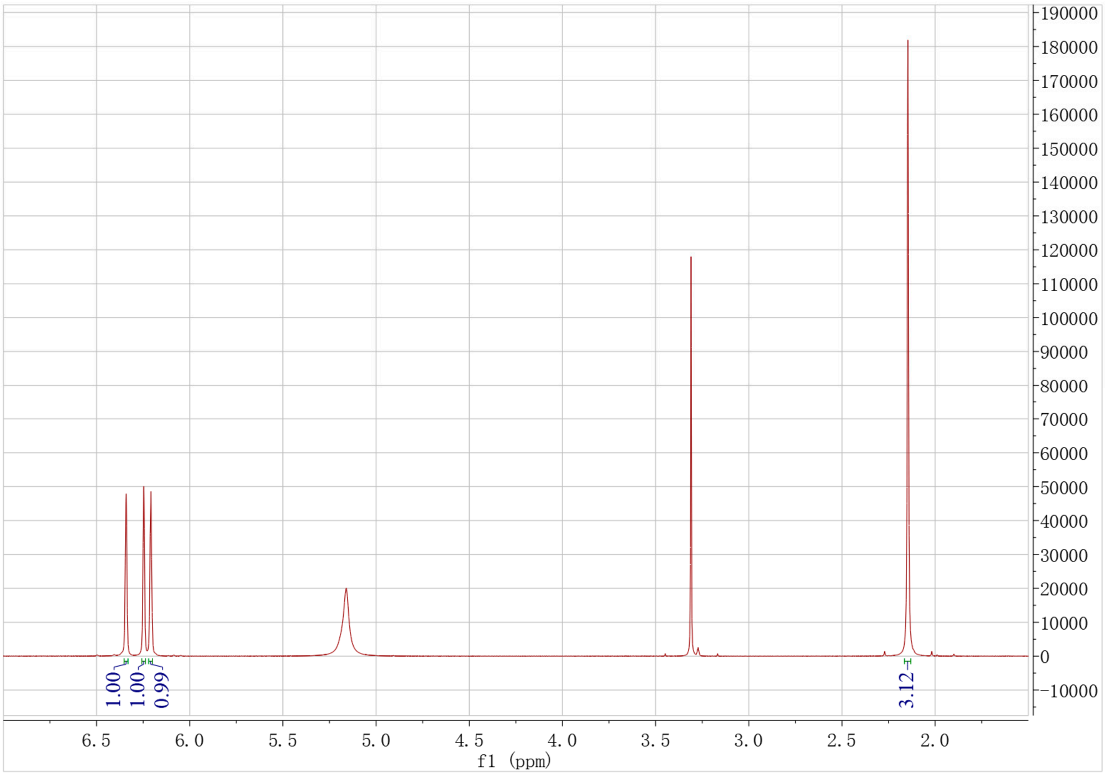

Figure S8. ^{1}H NMR (500 MHz, CD_3OD) spectrum of compound **4**.

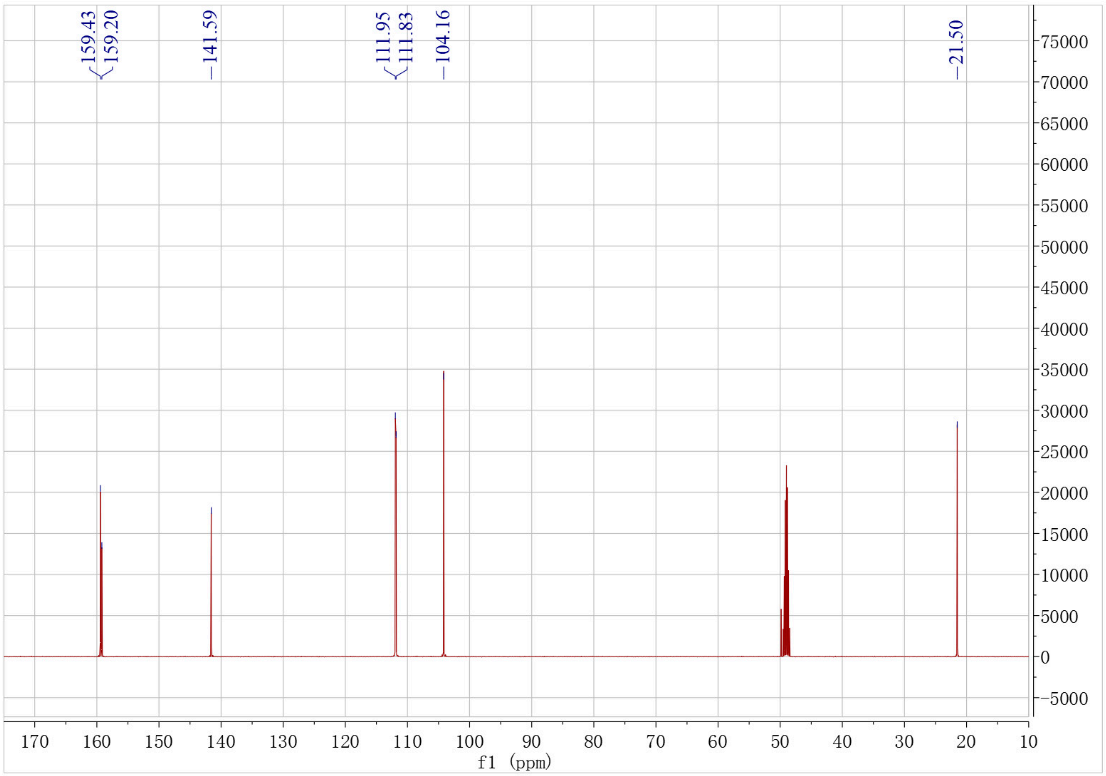

Figure S9. ^{13}C NMR (125 MHz, CD_3OD) spectrum of compound **4**.

Table S1. Soil physicochemical factors of three sampling zones.

Sampling sites		MC (%)	pH	EC (ms/cm)	TDS (g/kg)	TK (g/kg)	TP (g/kg)	TN (mg/kg)	SOM (g/Kg)
May	GD	25.38+0.86 a	8.15+0.19 c	6.59+0.27 a	7.43+0.20 a	16.70+0.56 d	0.56+0.02 a	1.20+0.03 a	12.52+0.40 d
	NT	22.56+2.35 ab	8.03+0.05 c	17.03+0.67 b	21.90+1.96 f	16.75+1.83 d	0.56+0.11 a	0.18+0.07 a	6.4+0.44 c
	NY	20.70+0.76 bc	8.22+0.33 c	6.72+0.20 a	7.86+0.52 ab	22.11+0.70 g	0.68+0.06 c	1.25+0.05 a	21.33+0.24 e
July	GD	23.52+1.24 ab	7.26+0.14 ab	7.78+1.36 a	14.03+0.74 d	14.29+0.43 bc	0.58+0.02 a	41.44+1.23 d	2.41+0.06 b
	NT	20.39+0.49 bc	7.24+0.11 a	8.47+1.94 a	15.02+0.66 d	20.45+0.58 f	0.7+0.12 c	57.83+1.04 e	2.71+0.09 b
	NY	25.77+1.13 a	7.33+0.16 ab	7.9+1.51 a	9.24+0.12 b	18.37+0.80 e	0.62+0.10 b	60.28+0.17 f	2.69+0.13 b
October	GD	22.16+1.99 ab	7.55+0.08 b	14.36+1.00 b	17.91+0.64 e	11.33+0.43 a	0.62+0.13 b	13.84+1.21 b	0.33+0.03 a
	NT	17.30+0.98 cd	7.48+0.10 ab	17.32+0.50 b	22.17+1.20 f	13.28+0.59 b	0.68+0.12 c	13.42+0.45 b	0.43+0.03 a
	NY	13.39+0.65 d	7.45+0.08 ab	9.73+0.15 a	11.73+0.67 c	15.36+0.23 cd	0.66+0.03 c	25.35+0.22 c	0.37+0.04 a

[a] Different lowercase letters in a column indicated the means were significantly different at $p < 0.05$ according to the Duncan's multiple range test.

Table S2. Alpha-diversity indices of three sampling zones.

Sampling sites		OTUs	Shannon	Simpson	Chao1	Ace
May	GD	301	3.017	0.136	392.244	457.295
	NT	23	1.838	0.328	23	0
	NY	496	3.868	0.047	527.318	525.889
July	GD	60	2.729	0.174	60	60
	NT	149	3.740	0.054	149	149
	NY	486	3.853	0.052	534.487	537.674
October	GD	30	1.856	0.228	45	54.879
	NT	166	3.284	0.073	167.667	167.912
	NY	56	2.535	0.170	58	59.862

Table S3. Isolation of cultivable rhizosphere fungi from three sampling zones and their classifications at the genus level.

Genus	May			July			October		
	GD	NT	NY	GD	NT	NY	GD	NT	NY
Acremonium	−	−	1	1	−	−	−	−	−
Alternaria	−	1	−	−	1	−	−	−	−
Apophysomyces	1	−	−	−	−	−	−	−	−
Aspergillus	8	−	2	5	−	1	1	−	−
Chrysosporium	1	−	−	−	−	−	−	−	−
Cladosporium	1	−	−	−	−	−	−	−	−
Corallomycetella	−	−	−	−	−	−	−	−	−
Dichotomopilus	1	−	−	−	−	−	−	−	−
Emericellopsis	−	−	1	−	−	−	−	−	−
Hypocreales	−	−	−	1	−	−	−	1	−
Mortierella	−	−	−	1	−	−	−	−	−
Neocamarosporium	1	1	−	−	−	−	−	−	−
Penicillium	3	−	2	3	−	−	1	−	−
Pleosporales	−	−	−	−	−	−	−	−	1
Purpureocillium	−	−	−	−	−	−	−	−	2
Saksenaea	−	−	−	−	−	−	−	−	−
Sarocladium	−	−	2	−	−	−	−	−	−
Sedecimiella	−	−	1	−	−	−	−	−	−
Talaromyces	−	−	4	−	−	2	−	1	−
Xenoacremonium	−	−	−	−	−	1	−	1	−

Table S4. Identification of cultivable rhizosphere fungi from three sampling zones and their antifungal and herbicidal potentials.

Strain number	Possible species	Similarity (%)	Inhibition rates (%)		
			B. cinerea	Roots of *E. crusgalli*	Stems of *E. crusgalli*
GD−1	*Chrysosporium* sp.	97.5	24.4	−	−
GD−2	*Aspergillus keveii*	100	28.0	27.0	33.7
GD−3	*Aspergillus jensenii*	100	31.2	72.3	67.1
GD−4	*Dichotomopilus indicus*	99.8	31.4	−	52.0
GD−5	*Penicillium terrigenum*	100	32.5	70.1	40.3
GD−6	*Neocamarosporium* sp.	96.1	33.7	−	−
GD−7	*Aspergillus tennesseensis*	100	35.1	−	−
GD−8	*Cladosporium antarcticum*	100	38.4	−	−
GD−9	*Hypocreales* sp.	99.8	41.2	75.2	67.3
GD−10	*Aspergillus sclerotiorum*	100	41.3	69.2	66.3
GD−11	*Aspergillus tabacinus*	100	41.9	−	−

Strain number	Possible species	Similarity (%)	Inhibition rates (%)		
			B. cinerea	Roots of *E. crusgalli*	Stems of *E. crusgalli*
GD−12	*Aspergillus tennesseensis*	100	43.0	−	−
GD−13	*Acremonium potronii*	100	45.9	30.8	30.6
GD−14	*Aspergillus tennesseensis*	100	46.7	32.5	23.2
GD−15	*Aspergillus sydowii*	100	47.7	−	−
GD−16	*Penicillium chrysogenum*	100	47.7	−	−
GD−17	*Aspergillus insuetus*	99.8	48.2	30.8	43.9
GD−18	*Aspergillus tennesseensis*	100	49.8	−	−
GD−19	*Aspergillus sydowii*	100	50.0	−	−
GD−20	*Penicillium* sp.	100	50.6	−	−
GD−21	*Penicillium kewense*	99.8	51.2	−	−
GD−22	*Penicillium terrigenum*	100	59.5	−	−
GD−23	*Apophysomyces* sp.	96.9	60.0	−	−
GD−24	*Penicillium oxalicum*	100	68.6	−	−
GD−25	*Aspergillus tabacinus*	100	82.6	99.3	99.0
GD−26	*Mortierella* sp.	98.1	−	32.5	23.2
GD−27	*Penicillium oxalicum*	100	−	−	−
GD−28	*Aspergillus insuetus*	100	−	−	−
GD−29	*Aspergillus sydowii*	100	−	−	−
NT−1	*Alternaria destruens*	100	34.7	−	−
NT−2	*Xenoacremonium* sp.	100	36.5	38.3	44.4
NT−3	*Talaromyces pinophilus*	100	40.0	−	−
NT−4	*Hypocreales* sp.	99.8	45.9	60.3	59.0
NT−5	*Neocamarosporium* sp.	96.1	47.2	−	−
NT−6	*Alternaria alternata*	100	−	−	−
NY−1	*Talaromyces columbinus*	100	20.0	−	−
NY−2	*Acremonium sclerotigenum*	100	23.5	−	−
NY−3	*Sarocladium terricola*	100	23.5	−	−
NY−4	*Emericellopsis atlantica*	99.6	28.1	−	46.1
NY−5	*Talaromyces albobiverticillius*	99.6	28.6	38.5	46.4
NY−6	*Sedecimiella alba*	99.8	41.9	−	38.9
NY−7	*Purpureocillium lilacinum*	100	43.5	38.3	41.0
NY−8	*Talaromyces fuscoviridis*	99.2	47.7	−	−
NY−9	*Talaromyces fuscoviridis*	99.2	47.7	48.5	−
NY−10	*Xenoacremonium falcatum*	100	48.2	−	−
NY−11	*Talaromyces fuscoviridis*	99.2	48.6	−	−
NY−12	*Aspergillus iizukae*	99.6	51.6	90.3	30.1
NY−13	*Aspergillus urmiensis*	100	52.8	−	−
NY−14	*Talaromyces fuscoviridis*	99.2	53.5	−	−
NY−15	*Penicillium brasilianum*	100	74.9	−	−
NY−16	*Sarocladium terricola*	100	75.6	−	−
NY−17	*Penicillium brasilianum*	100	78.8	−	−
NY−18	*Aspergillus tabacinus*	100	−	−	−
NY−19	*Pleosporales* sp.	100	−	−	−
NY−20	*Purpureocillium lilacinum*	100	−	70.1	40.3
